# Use of Biomarkers to Improve 28-Day Mortality Stratification in Patients with Sepsis and SOFA ≤ 6

**DOI:** 10.3390/biomedicines11082149

**Published:** 2023-07-30

**Authors:** Jaume Baldirà, Juan Carlos Ruiz-Rodríguez, Adolfo Ruiz-Sanmartin, Luis Chiscano, Alejandro Cortes, Diego Ángeles Sistac, Roser Ferrer-Costa, Inma Comas, Yolanda Villena, Maria Nieves Larrosa, Juan José González-López, Ricard Ferrer

**Affiliations:** 1 Intensive Care Department, Hospital de la Santa Creu i Sant Pau, 08041 Barcelona, Spain; jbaldira@santpau.cat (J.B.); dangeles@santpau.cat (D.Á.S.); 2 Department de Medicina, Universitat Autònoma de Barcelona, 08193 Barcelona, Spainricard.ferrer@vallhebron.cat (R.F.); 3 Intensive Care Department, Hospital Universitari Vall d’Hebron, Campus Vall d’Hebron, 08035 Barcelona, Spain; adolfo.ruiz@vallhebron.cat (A.R.-S.); alejocortes6@gmail.com (A.C.); 4 Shock, Organ Dysfunction and Resuscitation Research Group, Vall d’Hebron Institut de Recerca, Campus Vall d’Hebron, 08035 Barcelona, Spain; 5 Clinical Laboratories, Clinical Biochemistry Department, Vall d’Hebron University Hospital, 08035 Barcelona, Spain; roser.ferrer@vallhebron.cat (R.F.-C.); imma.comas@vallhebron.cat (I.C.); yolanda.villena@vallhebron.cat (Y.V.); 6 Microbiology Department, Vall d’Hebron University Hospital, 08035 Barcelona, Spain; nieves.larrosa@vallhebron.cat (M.N.L.); juanjo.gonzalez@vallhebron.cat (J.J.G.-L.); 7 Microbiology Research Group, Vall d’Hebron Institut de Recerca (VHIR), Vall d’Hebron Barcelona Hospital Campus, 08035 Barcelona, Spain; 8 Department of Genetics and Microbiology, Universitat Autònoma de Barcelona, 08193 Bellaterra, Spain; 9 CIBER de Enfermedades Infecciosas (CIBERINFEC), Instituto de Salud Carlos III, 28029 Madrid, Spain

**Keywords:** sepsis biomarkers, mortality, MR-proADM, SOFA score, lactate, molecular diagnosis

## Abstract

Early diagnosis and appropriate treatments are crucial to reducing mortality risk in septic patients. Low SOFA scores and current biomarkers may not adequately discern patients that could develop severe organ dysfunction or have an elevated mortality risk. The aim of this prospective observational study was to evaluate the predictive value of the biomarkers mid-regional pro-adrenomedullin (MR-proADM), procalcitonin (PCT), C-reactive protein (CRP), and lactate for 28-day mortality in patients with sepsis, and patients with a SOFA score ≤6. 284 were included, with a 28-day all-cause mortality of 8.45% (*n* = 24). Non-survivors were older (*p* = 0.003), required mechanical ventilation (*p* = 0.04), were ventilated for longer (*p* = 0.02), and had higher APACHE II (*p* = 0.015) and SOFA (*p* = 0.027) scores. Lactate showed the highest predictive ability for all-cause 28-day mortality, with an area under the receiver-operating characteristic curve (AUROC) of 0.67 (0.55–0.79). The AUROC for all-cause 28-day mortality in patients with community-acquired infection was 0.69 (0.57–0.84) for SOFA and 0.70 (0.58–0.82) for MR-proADM. A 2.1 nmol/L cut-off point for this biomarker in this subgroup of patients discerned, with 100% sensibility, survivors from non-survivors at 28 days. In patients with community-acquired sepsis and initial SOFA score ≤ 6, MR-proADM could help identify patients at risk of 28-day mortality.

## 1. Introduction

Sepsis remains a critical public health issue [1] and is widely recognized as a leading cause of global mortality [2,3,4]. Therefore, early diagnosis and effective, appropriate treatments are crucial to reducing mortality risk [5].

Various definitions and scoring systems have been developed to aid in screening and quick diagnosis. The Sequential Organ Failure Assessment (SOFA) score, a well-established and widely used tool, objectively stratifies the risk of multi-organ failure by considering variables from six organ systems [6]. In 2016, the Third International Consensus Conference (Sepsis-3) for sepsis definition considered an increase of two or more in the SOFA score as diagnostic criteria, replacing the previously used SIRS criteria [2,7,8].

SOFA score has been shown to be associated with mortality [2,7,9], and in those with initial low SOFA values less than six, mortality is lower than 10% [10,11]. However, even with a reported low mortality risk in patients with a SOFA ≤ 6, a subgroup of sepsis patients with a low SOFA score still experiences high mortality rates. It is crucial to identify this specific subgroup of patients with early sepsis and high mortality to initiate timely and appropriate treatment. Although score systems such as the qSOFA have been designed to screen for sepsis, they may lack the sensitivity required, which has led to the Surviving Sepsis Campaign 2021 guidelines recommending against using the qSOFA as a single-screening tool for sepsis [12]. Blood biomarkers may provide the extra information required to identify this subgroup of patients at high risk or those who are developing organ failure despite a low SOFA score. Hence, there is a need to identify biomarkers that may assist in mortality risk-stratification in patients with an initial low SOFA score. The combined use of mid-regional pro-adrenomedullin (MR-proADM), procalcitonin (PCT), and other commonly used biomarkers, such as C-reactive protein (CRP), Interleukin-6 (IL-6), and lactate, have been suggested in previous studies [5,7,13,14,15,16]. Nonetheless, MR-proADM has shown recent usefulness with better prognostic accuracy than CRP and APACHE [5], and it is also correlated with a 30-day mortality [7,17]. MR-proADM may also indicate risk for multiple organ failure in sepsis patients despite a low or moderate SOFA score [18], with its concentrations corresponding to endothelial permeability [19]. Moreover, its combination with PCT also improves the diagnosis of sepsis [20]. PCT and CRP have also been well-documented for their use in sepsis and in discriminating between infectious and non-infectious etiology. However, neither has shown strong evidence for mortality prediction [5].

Therefore, we hypothesized that the biomarkers mid-regional pro-adrenomedullin (MR-proADM), procalcitonin (PCT), C-reactive protein (CRP), and lactate can help identify 28-day mortality in patients with sepsis and SOFA score ≤ 6.

## 2. Materials and Methods

### 2.1. Study Design

The present study involved a prospective observational analysis. This is a single-center observational study of patients who met the criteria for the activation of the in-hospital sepsis code (ISC) [5,21], between April 2016 and July 2018. The study endpoint was 28-day all-cause mortality following enrollment.

### 2.2. Setting and Participants 

Patients for whom the attending physician triggered the in-hospital sepsis code (ISC) at Hospital Universitari Vall d’Hebron in the emergency department (ED), hospital wards, and intensive care unit (ICU) with a diagnosis of sepsis or septic shock and initial SOFA score ≤ 6 were enrolled consecutively from April 2016 to July 2018.

The inclusion criteria were as follows: adult patients ≥18 years of age presenting with either a suspected or documented infection and met at least one of the two sets of variables of the Vall d’Hebron University Hospital in-hospital sepsis code (ISC) [5,21]. The ISC variables include (1) an acute alteration in the level of consciousness not explained by other clinical conditions, (2) hyperthermia (axillary temperature > 38.3 °C), hypothermia (axillary temperature < 36.0 °C) and/or tachycardia (>110 beats per minute), tachypnea (>30 breaths per minute), or low oxygen saturation (SpO_2_ < 90%), as well as arterial hypotension (systolic arterial pressure < 90 mmHg, mean arterial pressure < 65 mmHg, or a decrease of >40 mmHg of baseline systolic arterial pressure).

Patients younger than 18 years, patients who were pregnant, or patients for who no blood sample could be obtained were excluded. Patients were admitted to the ICU or a relevant ward according to clinical criteria.

### 2.3. Variables and Data Sources

Following the activation of the sepsis code, relevant data, including patient comorbidities, demographics, site of admission (ED vs. ward), and the classification of sepsis as nosocomial or community-acquired, were prospectively collected in the database. Triage data, laboratory results, microbiology tests, and final clinical diagnosis were also recorded.

The SOFA and APACHE II scores were calculated retrospectively upon enrollment.

Sepsis code activation also triggered the collection of blood samples, which were stored in the Sepsis Bank of Vall d’Hebron University Hospital Biobank until analysis. The samples used in this project were provided by the Sepsis Bank of Vall d’Hebron University Hospital Biobank and complied with appropriate ethics approval. Routine biomarker measurements tests included PCT (chemiluminescent immunoassay (CLIA)), CRP (immune turbidimetric test), and L-lactate (enzymatic color test). MR-proADM was analyzed retrospectively. MR-proADM was tested by blood sampling from the central catheter, and samples were stored at −80 °C. Samples were batch tested using TRACE technology (Time-Resolved Amplified Cryptate Emission, KRYPTOR^®^ platform, Thermo Fisher, Hennigsdorf, Germany). MR-proADM results were unavailable to the corresponding physician throughout patient enrollment and hospitalization.

### 2.4. Statistical Methods

**Figure 1:**
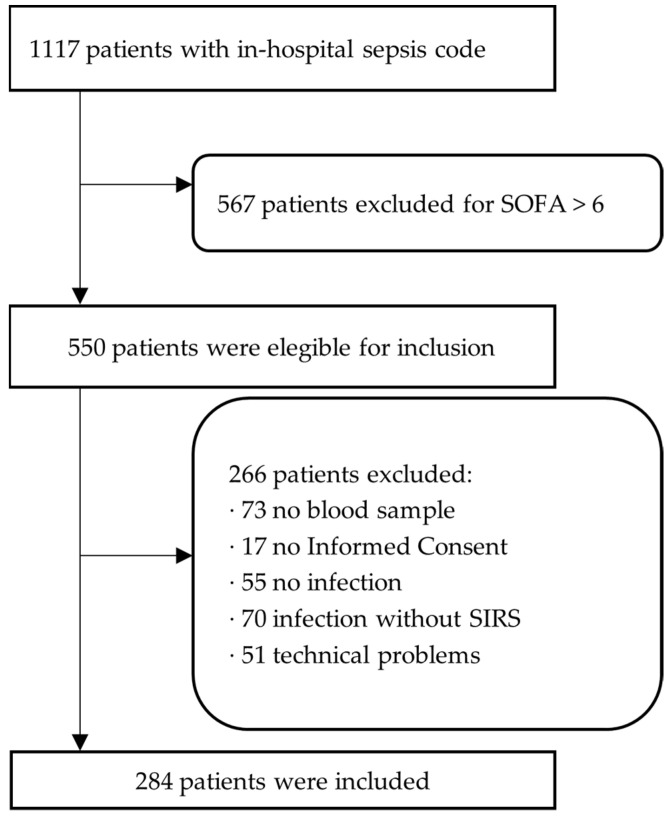
Flow chart of patients included in the present study.

To test our hypothesis, we estimated, using the Cochran formula, that an optimal sample of patients would be 300. During the study period, the in-hospital sepsis code was activated in 1117 patients. A total of 567 patients were excluded for presenting a SOFA score > 6. Of the remaining 550 patients, 284 were included in our final sample (Figure 1).

Descriptive data for continuous variables were displayed using mean and standard deviation; for discrete variables, median and the first to third quartile intervals were used, and categorical variables are represented in frequency and percentages. Variables were compared between surviving and non-surviving patients at 28 days following sepsis code activation. Differences were assessed using the chi-square test for categorical variables, Student’s *t*-test, and the Mann-Whitney U test for all other continuous and discrete variables.

The area under the receiver operating characteristic (AUROC) curves were used to evaluate each biomarker’s predictive ability and to identify the biomarker or clinical score with the greatest predictive value for the study endpoint. An AUROC of 0.5 was considered non-predictive, and 1.0 was considered a perfect predictive ability. An AUROC of 0.70 to 0.80 was considered acceptable [19].

The predictive performance of each indicator for mortality was assessed using univariate and multivariate logistic regression models. In addition, each indicator was also assessed for the following subgroups: 28-day mortality vs. 90-day mortality, nosocomial vs. community infection, medical vs. surgical patient, and ward v. ICU admission. Results are presented as odds ratio (OR) for mortality prediction with the corresponding 95% confidence intervals.

Kaplan–Meier survival analyses were performed, and Log-rank test results were included when analyzing MR-proADM usefulness to detect differences in survivability according to both AUROC results in this study and cut-off points previously reported in the pertaining literature. Finally, since we hypothesized that patients with a SOFA score ≤ 6 would have a particular biomarker profile, we conducted Pearson’s correlations to measure associations between MR-proADM, Lactate, PCT, and other clinical variables of interest. All reported *p*-values were two-sided and set with significance levels at <0.05. Statistical analysis was performed with both SPSS version 18.0 (IBM) and Stata version 12.0 (StataCorp LP, College Station, TX, USA).

## 3. Results

### 3.1. Participant Characteristics

A total of 284 patients were identified that met the inclusion criteria, with a 28-day all-cause mortality of 8.4% (N = 24). Of the 284 patients included, 184 (64.8%) patients had a diagnosis of sepsis, and 100 (35.2%) had septic shock. Patients had a mean age of 63 years, female patients accounted for 37.7%, and most cases were identified in the emergency department (48.6%). Positive blood cultures were obtained in 38.2% (*n* = 108) with predominantly Gram-negative bacteria growth (63.1%, *n* = 70). ISC was activated in 56 (19.7%) patients already admitted into the ICU. Of the remaining 228 patients, 62 (27.2%) were admitted into the ICU from the emergency department or another hospital ward. The median of the length of stay in the ICU was 4 days (2–8), and the median of the length of stay in the hospital was 13 (6–30) (Table 1).

### 3.2. Biomarker Predictive Ability in Patients with Sepsis and a SOFA Score ≤ 6

**Figure 2:**
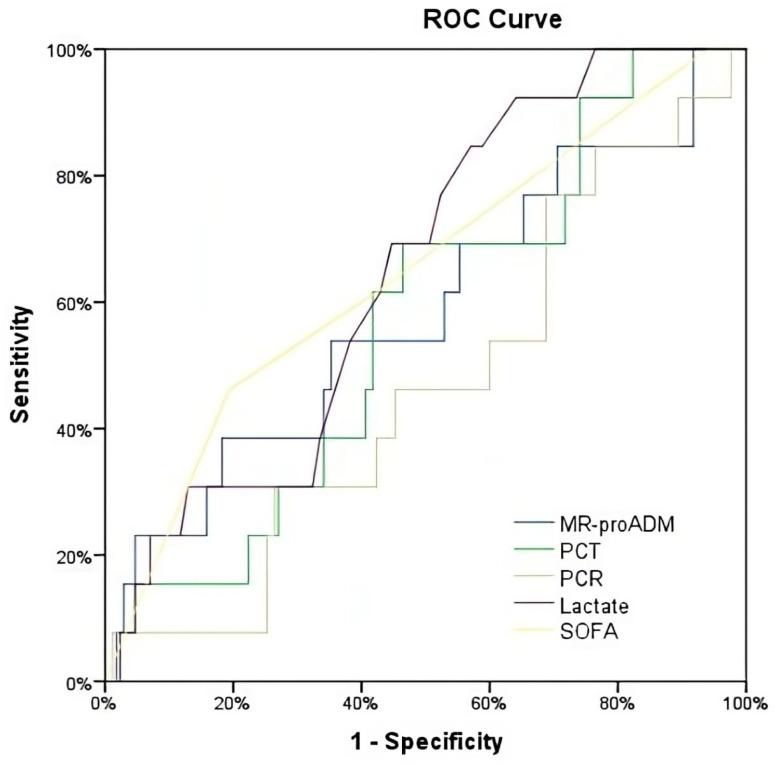
ROC for the prediction of mortality at 28 days.

**Figure 3:**
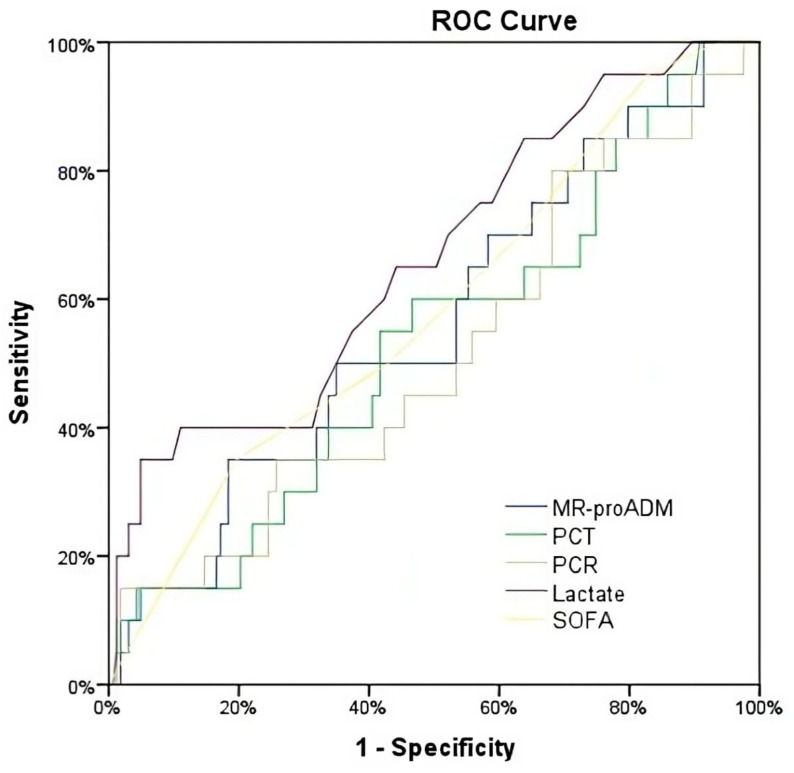
ROC for the prediction of mortality at 90 days.

Lactate showed the highest predictive ability for all-cause 28-day mortality with an AUROC of 0.67 (0.55–0.79). In contrast, the AUROC for MR-proADM was low, with an AUROC of 0.57 (0.45–0.69). Parallelly, the AUROC for CRP was 0.49 (0.36–0.62), and for PCT, it was 0.47 (0.35–0.59) (Table 2, Figure 2 and Figure 3).

Both the univariate and multivariate logistic regression analyses of mortality at 28 days by each and all biomarkers were not statistically significant; however, the SOFA score had a significant OR (95% CI) of 1.43 for the same endpoint (Table 2).

When considering 90-day all-cause mortality, the AUROC for all biomarkers showed similar AUROCs to those established for 28-day mortality. In the same manner, univariate and multivariate ORs of all biomarkers and SOFA scores were not statistically significant (Table 2).

The predictive ability of each biomarker for 28-day mortality was assessed individually for either community-acquired or hospital-acquired infections. In patients with community-acquired infection, the best predictive AUROCs corresponded to MR-proADM (0.70 [0.58–0.82]) and SOFA score (0.69 [0.54–0.84]). In contrast, for hospital-acquired infection, only lactate showed a similarly high AUROC (0.70 [0.57–0.87]). Nonetheless, univariate and multivariate ORs of all biomarkers and SOFA score were not statistically significant (Table 3).

### 3.3. Inter-Biomarker Associations and 28-Day Survival Analysis in Patients with Sepsis and a SOFA Score ≤ 6

**Figure 4:**
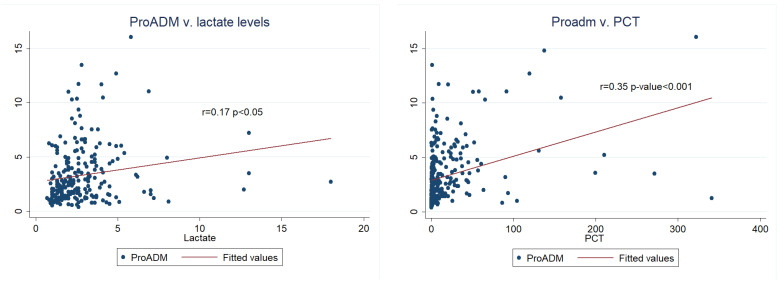
Correlation between MR-proADM and lactate and MR-proADM and PCT.

We found positive and significant correlations between MR-proADM, lactate, and PCT (Figure 4). Interestingly, from this secondary analysis, we also found a positive correlation between MR-proADM and age (r = 0.12, *p* < 0.05). Additionally, renal SOFA score was calculated, and we found a positive significant correlation with MR-proADM (r = 0.43, *p* < 0.0001).

**Figure 5:**
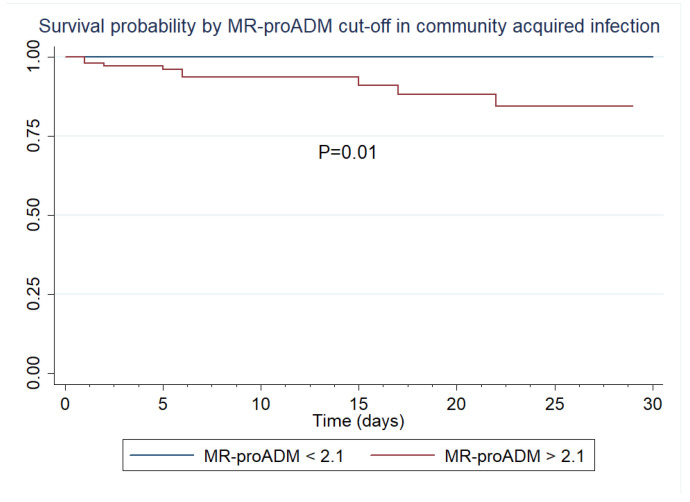
Kaplan–Meier survival curves by MR-proADM in community-acquired infection.

Additionally, according to our best predictive AUROC, we established that an MR-proADM cut-off point of 2.1 nmol/L classified, with 100% sensitivity, all non-survivors in patients with community-acquired infection. We generated a survival analysis for 28 days for this subgroup of patients using the 2.1 nmol/L MR-proADM cut-off. A Log-rank test of the Kaplan–Meier survival curves found significant statistical differences between survivors and non-survivors (Figure 5).

## 4. Discussion

The present study analyzed the predictive ability of various biomarkers for 28-day all-cause mortality in sepsis patients with a SOFA score ≤ 6. The overall mortality rate observed in this study was 8.45%, aligning with the mortality rates reported in the existing literature on this particular subject [11].

MR-proADM showed an acceptable AUROC for 28-day mortality prediction in sepsis patients with community-acquired infection. In contrast, lactate showed the highest predictive ability in sepsis patients with hospital-acquired infection. Other biomarkers showed low predictive ability in sepsis patients with a SOFA ≤ 6.

Scoring systems and clinical and laboratory variables as tools to aid in early diagnosis and to determine severity are well-established, and many biomarkers have been extensively studied and implemented in ICUs and emergency departments [5,23,24,25,26,27,28]. The SOFA score is widely applied and provides a screening score that indicates the risk of a poor outcome [29]. However, this score is not without inherent limitations and may not always be easy to apply [30,31,32]. Moreover, despite the low mortality risk in sepsis patients presenting with a SOFA < 6 [10], a subgroup of patients still appears to have a high mortality rate. Identifying this subgroup of patients may allow for the early initiation of appropriate treatment strategies, avoiding over- or under-treatment and reducing mortality [33]. Interestingly in the present study, even in patients with a SOFA ≤ 6, SOFA remained the best individual predictor of survival. Thus, even in the lower SOFA range, organic dysfunction still discriminated severity better than the inflammatory response or hypoperfusion measured by biomarkers. Nonetheless, recent studies have indicated the potential of novel biomarkers such as MR-proADM and PCT [5,34].

MR-proADM has been proposed as a potentially useful early marker in critically ill patients, as its concentrations correspond to microcirculatory and endothelial damage in the early stages of organ dysfunction [35,36,37,38]. Hence, it is considered an indicator of early microcirculatory damage in patients with a low SOFA score. Previous studies support this consideration, showing that MR-proADM may be elevated in those sepsis patients likely to develop multiple organ failure even with a low or moderate SOFA score [17]. Our results are in line with these affirmations, as inflammatory biomarkers are correlated with each other even with low SOFA scores.

Other authors have reported that MR-proADM indicates the development of organ failure 24 h in advance [39]. Furthermore, even with a SOFA score that does not fulfill the criteria for sepsis, an elevated MR-proADM identifies septic patients, allowing for early treatment initiation [7]. Parallelly, in this study, elevated MR-proADM levels correlated with age increase, a finding that has not been thoroughly investigated and that may lead to age-dependent cut-off points, increasing the diagnostic accuracy of this inflammatory biomarker. MR-proADM has also shown good predictability in identifying patients that would require ICU admission and had good accuracy in identifying 28-day and 90-day mortality [5].

The current study results are in line with previous studies and provide further supportive evidence for the consideration of its use as part of a panel of early biomarkers in sepsis patients. These results support those found by Elke et al. [17] and Andaluz-Ojeda et al. [40], with MR-proADM showing a reasonable AUROC for 28-day mortality in patients with a SOFA ≤ 6. Although the study by Andaluz-Ojeda et al. reported a higher AUROC for 28-day mortality for MR-proADM, their study only included ICU patients. In contrast, the present study included all hospital patients that activated the sepsis code regardless of their ward.

Moreover, in our study, a MR-proADM cut-off point of 2.1 nmol/L had 100% sensibility to discern non-survivors at 28 days for patients with community-acquired infection, making MR-proADM a potentially useful screening biomarker for severity in out-of-hospital sepsis.

Our results align with those of Spoto et al. [7,20], who regarded MR-proADM as crucial for early sepsis diagnosis in those with a negative SOFA score. Hence, these studies, along with the results of this study, point to the potential of MR-proADM to guide clinical decisions and stratify risk, including in those with less-severe disease or early sepsis.

Biomarkers may vary based on whether the infection is nosocomial or has its origin in the community. In particular, hospital-acquired sepsis patients were originally admitted for another cause that can affect these biomarkers—for example, major surgery, or other major illness—and may confound the results. In contrast, in community-acquired sepsis patients, any changes in biomarker values are most likely to represent only the effect of sepsis. Along these lines, the present study showed that MR-proADM might be more useful to predict mortality in community-acquired infections, while lactate may be better to forecast mortality for hospital-acquired infections.

### Limitations

The results of this study should be interpreted considering some limitations. First, the relatively low number of enrolled patients in a single center may have resulted in many subgroups being underpowered. Further, larger studies are needed to build on this study and determine the usefulness of the biomarkers studied. Second, the present study also reflected real clinical practice, and we cannot ensure that during the study period the in-hospital sepsis code was activated in all patients with sepsis. Nevertheless, our results are consistent with those previously discussed in the literature [16,40], despite the patient population enrolled in this study being preselected based on fulfilling a particular hospital sepsis criterion. Hence, this study provides additional supportive data for these biomarkers and their use at different hospital settings and varying levels of illness.

## 5. Conclusions

The findings of the present study suggest that, in patients with sepsis acquired in the community and an initial SOFA score ≤ 6, MR-proADM could help identify patients at risk of 28-day mortality. In those with hospital-acquired infection, lactate provided better predictive ability for 28-day mortality. Therefore, the initial measurement of these biomarkers could aid in implementing early treatment strategies based on risk stratification after activation of a hospital sepsis code in patients with a SOFA ≤ 6 and sepsis or septic shock.

## Figures and Tables

**Table 1:** Clinical patient characteristics upon activation of the sepsis code with respect to the total infected patient population and subsequent 28-day mortality.

	Patient Population (*N* = 284)	Survivors (*n* = 260)	Non-Survivors (*n* = 24)	*p*-Value
Age (years) (mean, S.D.)	63 (16)	62 (16)	70 (9)	0.001
Female gender (*N*, %)	107 (37.7)	100 (38.5)	7 (29.2)	0.36
Definition of sepsis				
Severe sepsis (*N*, %)	184 (64.8)	170 (65.4)	14 (58.3)	0.489
Septic shock (*N*, %)	100 (35.2)	90 (34.6)	10 (41.7)	0.489
Location of sepsis code activation				
Emergency department (*N*, %)	138 (48.6)	127 (48.8)	11 (45.8)	0.793
Ward (*N*, %)	90 (31.7)	83 (31.9)	7 (29.2)	0.781
ICU (*N*, %)	56 (19.7)	50 (19.2)	6 (25)	0.497
Surgical admissions (*N*, %)	104 (36.6)	95 (36.5)	9 (37.5)	0.925
Medical admissions (*N*, %)	180 (63.4)	165 (63.5)	15 (62.5)	0.925
ICU length of stay (days) (median, IQR)	4 [2–9]	3 [2–8]	11 [5–15]	0.125
Hospital length of stay (days) (median, IQR)	13 [6–30]	14 [7–32]	7 [3–17]	0.016
Life-supporting and intensive care therapies				
Vasopressors (*N*, %)	100 (35.2)	90 (34.6)	10 (41.7)	0.489
Renal replacement therapy (*N*, %)	11 (8.5)	9 (7.5)	2 (20)	0.172
Mechanical ventilation (*N*, %)	51 (18)	43 (16.5)	8 (33.3)	0.04
Mechanical ventilation duration (days) (median, IQR)	5 [3–12]	4 [2–8]	12 [7–16]	0.02
High-flow nasal cannula use (*N*, %)	41 (31.5)	35 (29.2)	6 (60)	0.044
Pre-existing comorbidities				
Cardiopathy (*N*, %)	71 (25)	65 (25)	6 (25)	1.00
Chronic kidney disease (*N*, %)	50 (17.6)	47 (18.1)	3 (12.5)	0.492
COPD (*N*, %)	49 (17.3)	45 (17.3)	4 (16.7)	0.937
Immunosuppression (*N*, %)	129 (45.4)	112 (43.1)	17 (70.8)	0.009
Liver cirrhosis (*N*, %)	12 (4.2)	9 (3.5)	3 (12.5)	0.035
Microbiology				
Positive blood culture (*N*, %)	108 (38.2)	99 (38.2)	9 (37.5)	0.622
Gram-positive (*N*, %)	39 (35.1)	34 (33.3)	5 (55.6)	0.291
Gram-negative (*N*, %)	70 (63.1)	66 (64.7)	4 (44.4)	0.343
Fungal (*N*, %)	2 (1.8)	2 (2)	0 (0)	0.666
Origin of infection				
Abdominal (*N*, %)	76 (26.8)	72 (27.7)	4 (16.7)	0.243
Bacteria-primary (*N*, %)	11 (3.9)	10 (3.8)	1 (4.2)	0.938
Catheter-related (*N*, %)	11 (3.9)	10 (3.8)	1 (4.2)	0.938
Central nervous system (*N*, %)	1 (0.4)	1 (0.4)	0 (0)	0.761
Respiratory (*N*, %)	68 (23.9)	57 (21.9)	11 (45.8)	0.009
Soft-tissue (*N*, %)	18 (6.3)	16 (6.2)	2 (8.3)	0.675
Urinary (*N*, %)	84 (29.6)	79 (30.4)	5 (20.8)	0.327
Unknown (*N*, %)	8 (2.8)	8 (3.1)	0 (0)	0.383
Other (*N*, %)	7 (2.5)	7 (2.7)	0 (0)	0.416
Source control				
Debridement of infectious foci (*N*, %)	15 (5.4)	14 (5.5)	1 (4.3)	0.799
Drainage (*N*, %)	40 (14.4)	38 (14.9)	2 (8.7)	0.397
Surgery (*N*, %)	35 (12.6)	33 (12.9)	2 (8.7)	0.534
Biomarker and severity scores				
Leucocytes (x10E9/L) (mean, SD)	13.82 (8.88)	13.79 (8.88)	14.09 (9.46)	0.438
Platelets (x10E9/L) (mean, SD)	235 (148)	236 (146)	228 (166)	0.600
MR-proADM (nmol/L) (median, IQR)	2.48 [1.46–4.38]	2.43 [1.45–4.2]	2.85 [1.8–4.98]	0.236
PCT (ng/mL) (median, IQR)	3.09 [0.7–16.1]	3.10 [0.7–17.5]	3.51 [0.7–8.8]	0.665
Lactate (mmol/L) (mean, SD)	2.75 (2.09)	2.70 (2.10)	3.48 (1.84)	0.147
CRP (mg/dL) (mean, SD)	56.43 (288.39)	46.18 (258.14)	156.57 (494)	0.312
SOFA (points) (median, IQR)	4 [3–6]	4 [3–6]	5 [4–6]	0.027
APACHE II (points) (mean, SD)	19.71 (8.29)	19.10 (8.11)	26.50 (7.61)	0.015

**Table 2:** Area under the ROC for the prediction of mortality at 28 days and 90 days.

				Analyses	
				Univariate	Multivariate
	Biomarker or Clinical Score	Patients (N)	AUROC	OR IQR (95% CI)	OR IQR (95% CI)
M28d	MR-proADM	284	0.57 [0.45–0.69]	1.08 [0.94–1.24]	1.13 [0.82–1.56]
	PCT	274	0.47 [0.35–0.59]	0.99 [0.98–1.01]	0.99 [0.97–1.02]
	CRP	237	0.49 [0.36–0.62]	1.00 [0.99–1.01]	1.01 [0.98–1.04]
	Lactate	229	0.67 [0.55–0.79]	1.13 [0.95–1.34]	0.85 [0.49–1.49]
	SOFA	284	0.63 [0.52–0.73]	1.43 [1.03–1.97]	1.63 [0.69–3.84]
M90d	MR-proADM	284	0.59 [0.49–0.68]	1.08 [0.96–1.21]	0.93 [0.72–1.20]
	PCT	274	0.44 [0.34–0.54]	1.00 [0.99–1.00]	0.99 [0.97–1.01]
	CRP	237	0.54 [0.43–0.65]	1.00 [0.99–1.01]	1.00 [0.98–1.03]
	Lactate	229	0.66 [0.55–0.77]	1.25 [1.07–1.46]	1.50 [0.90–2.50]
	SOFA	284	0.62 [0.54–0.71]	1.40 [1.08–1.81]	1.31 [0.70–2.43]

Abbreviations: AUROC, area under the ROC; CI, confidence interval; OR, odds ratio; IQR, interquartile range; M28d, 28-day mortality; M90d, 90-day mortality. Multivariate analyses show the OR for predictive ability of every biomarker when combined between themselves and the inclusion of other variables: age, SOFA, APACHE II, and nosocomial infection.

**Table 3:** AUROCs and ORs for 28-day mortality in hospital-acquired vs. community-acquired infections.

				Analyses	
				Univariate	Multivariate
	Biomarker or Clinical Score	Patients (N)	AUROC	OR IQR (95% CI)	OR IQR (95% CI)
Community	MR-proADM	176	0.70 [0.58–0.82]	1.11 [0.91–1.35]	2.11 [0.7–6.39]
	PCT	168	0.41 [0.30–0.52]	0.92 [0.82–1.04]	0.93 [0.79–1.11]
	CRP	147	0.55 [0.32–0.78]	1.01 [1.00–1.03]	1.01 [0.83–1.25]
	Lactate	142	0.65 [0.48–0.82]	1.09 [0.75–1.56]	1.31 [0.85–20.20]
	SOFA	176	0.69 [0.54–0.84]	1.80 [0.98–3.30]	-
Hospital	MR-proADM	108	0.48 [0.30–0.66]	1.07 [0.88–1.30]	1.00 [0.69–1.46]
	PCT	106	0.53 [0.35–0.71]	1.00 [0.99–1.00]	1.01 [0.96–1.06]
	CRP	87	0.49 [0.32–0.66]	0.99 [0.98–1.01]	1.00 [0.95–1.04]
	Lactate	93	0.70 [0.54–0.86]	1.14 [0.93–1.38]	0.86 [0.44–1.65]
	SOFA	108	0.57 [0.43–0.71]	1.23 [0.85–1.77]	1.18 [0.54–2.59]

Abbreviations: AUROC, area under the ROC; CI, confidence interval; OR, odds ratio; IQR, interquartile range; M28d, 28-day-mortality; M90d, 90-day mortality. Multivariate analyses show the OR for the predictive ability of every biomarker when combined between themselves and the inclusion of SOFA and APACHE II scores.

## Data Availability

The datasets used and/or analyzed during the present study are available from the corresponding author upon reasonable request.
